# Altered phenotype and Stat1 expression in Toll-like receptor 7/8 stimulated monocyte-derived dendritic cells from patients with primary Sjögren’s syndrome

**DOI:** 10.1186/ar4682

**Published:** 2014-08-11

**Authors:** Petra Vogelsang, Marie Karlsen, Johan G Brun, Roland Jonsson, Silke Appel

**Affiliations:** Broegelmann Research Laboratory, Department of Clinical Science, University of Bergen, Jonas Lies vei 87, N-5021 Bergen, Norway; Department of Rheumatology, Haukeland University Hospital, PB 1400, N-5021 Bergen, Norway; Department of Clinical Science, University of Bergen, Jonas Lies vei 87, N-5021 Bergen, Norway

## Abstract

**Introduction:**

Dendritic cells (DC) are the most potent antigen-presenting cells of the immune system, involved in both initiating immune responses and maintaining tolerance. Dysfunctional and via toll-like receptor (TLR) ligands activated DC have been implicated in the development of autoimmune diseases, but their role in the etiology of Sjögren’s syndrome, a chronic inflammatory autoimmune disease characterized by progressive mononuclear cell infiltration in the exocrine glands, has not been revealed yet. Therefore, the aim of this study was to investigate phenotype and functional properties of immature and TLR7/8 stimulated monocyte-derived DC (moDC) of patients with primary Sjögren’s syndrome (pSS) and compare them to healthy controls.

**Methods:**

The phenotype, apoptosis susceptibility and endocytic capacity of moDC were analyzed by flow cytometry. Secretion of cytokines was measured by enzyme-linked immunosorbent assay (ELISA) and multiplex Luminex analyses in moDC cell culture supernatants. The expression of TLR7 was analyzed by flow cytometry and real-time quantitative polymerase chain reaction (qPCR). Expression of Ro/Sjögren’s syndrome-associated autoantigen A (Ro52/SSA), interferon regulatory factor 8 (IRF-8), Bim, signal transduction and activators of transcription (Stat) 1, p-Stat1 (Tyrosin 701), p-Stat1 (Serin 727), Stat3, pStat3 (Tyrosin 705) and glyceraldehyde 3-phosphatase dehydrogenase (GAPDH) was measured by Western blotting. Nuclear factor kappa-light-chain-enhancer of activated B cells (NF-κB) family members were quantified using the ELISA-based TransAM NF-κB family kit.

**Results:**

We could not detect differences in expression of co-stimulatory molecules and maturation markers such as cluster of differentiation (CD) 86, CD80, CD40 or CD83 on moDC from patients compared to healthy controls. Moreover, we could not observe variations in apoptosis susceptibility, Bim and Ro52/SSA expression and the endocytic capacity of the moDC. However, we found that moDC from pSS patients expressed increased levels of the major histocompatibility complex (MHC) class II molecule human leukocyte antigen (HLA)-DR. We also found significant differences in cytokine production by moDC, where increased interleukin (IL)-12p40 secretion in mature pSS moDC correlated with increased RelB expression. Strikingly, moDC from pSS patients matured for 48 hours with TLR7/8 ligand CL097 expressed significantly less Stat1.

**Conclusion:**

Our results suggest a role for moDC in the pathogenesis of Sjögren’s syndrome.

## Introduction

Sjögren’s syndrome (SS) is a chronic, inflammatory autoimmune disease characterized by progressive mononuclear cell infiltration in the salivary and lacrimal glands of mainly T cells but also B cells and dendritic cells (DC) [1]. Patients with primary Sjögren’s syndrome (pSS) suffer from dryness of the mouth (xerostomia) and eyes (keratoconjunctivitis sicca), whereas in patients with secondary SS the symptoms occur in combination with other related rheumatic autoimmune diseases.

Serological characteristics of systemic autoimmune diseases such as SS are the production of autoantibodies and a skewed profile of several cytokines. Notably, type I interferon (IFN) alpha has been strongly related to SS and systemic lupus erythematosus (SLE) [2], and upregulation of IFN-regulated genes in salivary glands [3–5] and monocytes [6] has been described previously in patients with SS. In response to IFN, the transcription factor Stat1 becomes activated via phosphorylation on tyrosine (Y701) and serine (S727) residues and directs transcription of IFN-regulated genes [7].

Intriguingly, many autoantibodies associated with autoimmune diseases are directed towards cellular nuclear components. Defective binding of self-molecules to toll-like receptor (TLR) and inadequate activation of antigen-presenting cells (APCs) might thus be the trigger for the aberrant immune activation seen in autoimmune patients [8]. In patients with SS, frequently found autoantibodies are directed towards Ro/SSA, consisting of Ro52/SSA and Ro60/SSA, as well as La/SSB. These autoantibodies can even be detected in many patients long before the onset of symptoms [9]. Ro52/SSA has been identified as an E3 ubiquitin ligase, which among others can ubiquitinate members of the interferon regulatory factor (IRF) family [10, 11]. One target of Ro52/SSA was found to be IRF-8, a transcription factor that is important for both the development of DC and expression of cytokines such as IFN [12, 13]. IRFs become activated after stimulation with TLR ligands, and thus autoantibodies against Ro52/SSA might indirectly influence the transcription of both type I IFN and IFN-inducible genes. This might be of importance as several studies have suggested a central role for DC and type I IFN in the pathogenesis of autoimmune diseases [14].

DC are key players in both initiating an immune response and maintaining tolerance [15, 16]. During the development from antigen-capturing cells to APCs, DC undergo a maturation process characterized by morphological and phenotypical changes, involving loss of endocytic capacity as well as upregulation of molecules such as CD40, CD80, CD83 and CD86, which are important molecules for priming of naïve T cells [17].

DC comprise a heterogeneous population of cells, which in humans is divided into two main subsets, myeloid DC and the type I IFN-producing plasmacytoid DC [18, 19]. DC populations are relatively rare in blood, but *in vitro* generated monocyte-derived dendritic cells (moDC) are functional APCs that also develop from monocytes under inflammatory conditions in the body and thus are a useful tool to study DC functions *ex vivo*[20].

The breakdown of peripheral tolerance in autoimmune patients might be a result of defective DC. In patients with SLE, DC have been shown to overexpress CD86 and might therefore be overactive in stimulating T cells [21, 22]. Furthermore, the lifespan and apoptosis susceptibility of DC might influence the duration of activating T cells and consequently controlling immune responses, as transgenic mice with DC defective in apoptosis eventually develop systemic autoimmune manifestations [23, 24]. In particular, the proapoptotic protein Bim and the antiapoptotic protein Bcl-2 have been at the center of several investigations since they have been shown to control the lifespan of DC [24, 25]. Despite their pivotal role in tolerance maintenance, few functional and phenotypical studies have so far been performed with DC from patients with pSS. It has been proposed that an initial viral infection might trigger the onset of autoimmune diseases by increasing cytokine production in response to TLR activation [26]. We therefore chose the TLR7/8 ligand CL097 as a maturation stimulus in our study, because it mimics viral single-stranded RNA components.

The aim of this study was to investigate possible defects in moDC of patients with pSS. Our results showed that these cells are phenotypically similar and have a normal endocytic capacity compared with moDC from healthy controls, although they express higher amounts of surface HLA-DR and CCR7 molecules. Moreover, these cells differ from normal control cells in secretion of several cytokines (interleukin (IL)-7, IL-12p40, tumor necrosis factor alpha (TNFα), IFN gamma and macrophage inflammatory protein (MIP)-1α). Elevated IL-12p40 secretion correlated with increased nuclear RelB expression in cells stimulated for 48 hours with TLR7/8 ligand CL097. Additionally, mature moDC from pSS patients had significant lower proportions of Stat1 and p-Stat1 compared with healthy controls, suggesting that moDC might contribute to the pathogenic events seen in patients with pSS.

## Methods

### Patient samples

In total, 52 pSS patients and 55 controls were included in this study (Table 1). Owing to restricted numbers of moDC generated from limited amounts of fresh blood obtained for patients and controls, not all experiments could be performed for all samples. Numbers of samples used for the individual experiments are indicated in the figure legend. All patients fulfilled the American–European Consensus group criteria for classification of SS [27] and were recruited from the Rheumatology Clinic at the Haukeland University Hospital, Bergen, Norway. Gender-matched and age-matched healthy controls were obtained from the Bloodbank at the Haukeland University Hospital. The study was approved (# 242.06) by the ethical committee of the University of Bergen, Norway. All studied subjects gave their informed consent.

**Table 1:** **Characteristics of patient and control cohort used in this study**

	Sjögren’s syndrome	Healthy controls
Number	52	55
Age (years)		
Mean ± standard deviation	58 ± 13	52 ± 8
Range	20 to 81	38 to 68
Female:male	51:1	53:2

### Preparation and maturation of monocyte-derived dendritic cells

For generation of moDC, 20 to 45 ml heparinized blood were collected from each participant and handled within 2 hours. Peripheral blood mononuclear cells were isolated by discontinuous gradient centrifugation using Lymphoprep (Axis Shield PoC AS, Oslo, Norway) and monocytes isolated by plastic adherence in X-VIVO 20 (Lonza, Verviers, Belgium). Nonadherent cells were removed after 1 hour of incubation at 37°C and 5% carbon dioxide. Remaining monocytes were cultured in RP10 medium (RPMI 1640 with Ultraglutamine (Bio Whittaker, Lonza), 10% FCS Gold (PAA, Pasching, Austria), 1% penicillin–streptomycin (Gibco, Invitrogen Corporation, Paisley, UK)) supplemented with 100 ng/ml granulocyte–macrophage colony-stimulating factor (GM-CSF; ImmunoTools GmbH, Friesoythe, Germany) and 20 ng/ml IL-4 (ImmunoTools GmbH). Cytokines were replenished every 2 or 3 days. After 5 to 6 days in culture, maturation was induced in one part of the moDC by adding 1 μg/ml TLR7/8 ligand CL097 (InvivoGen, San Diego, CA, USA) for 48 hours.

### Immunostaining

Phenotypic characterization of moDC was performed using mouse anti-human CD14-fluorescein isothiocyanate (FITC), CD1a-phycoerythrin (PE), CD86-FITC and CD80-APC antibodies (all from ImmunoTools GmbH), HLA-DR-APC, CD83-PE, CD40-FITC and CD38-APC antibodies (all from AbD Serotec, Düsseldorf, Germany), and CCR7-PE and TLR7-PE antibodies (R&D Systems, Minneapolis, MN, USA). Detection of apoptosis was performed using Annexin V–FITC (ImmunoTools GmbH) for labeling of early apoptotic events and 7-AAD (eBioscience, San Diego, CA, USA) for discrimination of necrotic and dead cells. Cells were analyzed on a BD LRS Fortessa flow cytometer (BD Bioscience, San Jose, CA, USA) and data analysis was performed with FlowJo software (Tree Star Inc., Ashland, OR, USA). Both percentages of positive cells and the median fluorescence intensity (MFI) in the control sample were subtracted from either percentages of positive cells or the MFI in the stained sample.

### Cytokine production

Cytokine concentrations were measured in cell-free supernatants from immature and mature moDC cultures using a commercially available human cytokine twenty-five-plex kit (Biosource, Invitrogen Corporation, Carlsbad, CA, USA) including IL-1β, IL-1RA, IL-2, IL-2R, IL-4, IL-5, IL-6, IL-7, IL-8, IL-10, IL-12p40, IL-13, IL-15, IL-17, TNFα, IFN alpha, IFN gamma, GM-CSF, MIP-1α, MIP-1β, IP-10, MIG, Eotaxin, CCL5 (RANTES) and MCP-1. Multiplex plates were analyzed using a Luminex 100 instrument with StarSection software (Applied Cytometry Systems, Dinnington, UK). B-cell activating factor (BAFF) levels were measured using the human BAFF/BLys/TNFSF13B Quantikine enzyme-linked immunosorbent assay (ELISA) Kit from R&D Systems. IL-4 and GM-CSF in diluted samples as well as undiluted IL-12p70 levels were determined using ELISA *Deluxe* kits from Biolegend (San Diego, CA, USA) according to the manufacturer’s manual. IFN alpha was measured using the Verikine human IFN alpha multi-subtype ELISA kit (PBL Interferon Source, Piscataway, NJ, USA). All microtiter plates were analyzed with an EMax microplate reader (Molecular Devices, Sunnyvale, CA, USA).

### Real-time quantitative polymerase chain reaction

Total RNA was isolated from cell lysates using QIAGEN RNeasy Mini anion-exchange spin columns (QIAGEN, Hilden, Germany) according to the instructions of the manufacturer. Then 100 ng total RNA were subjected to a 20 μl cDNA synthesis reaction using RevertAid Reverse Transcriptase (Fermentas/Thermo Scientific, Oslo, Norway). Oligo(dT)_18_ was used as primer. The cDNA was diluted 1:2.5, and 5 μl were used in a 20 μl real-time quantitative reverse transcriptase polymerase chain reaction (PCR) using Taqman technology on an ABI PRISM 7500 Sequence Detection System (Applied Biosystems/Invitrogen Dynal AS, Oslo, Norway). The primers/probes utilized were: TLR7-FP, 5′-TGAATCTGTCAGGAAATCTCATTAGC; TLR7-RP, 5′-CAAGCCGGTTGTTGGAGAAG; TLR7 probe, 5′-[6FAM] CAGTGAATTCCAACCTTTAGCAGAGCTGAGATATT[BHQ1]; glyceraldehyde 3-phosphatase dehydrogenase (GAPDH)-FP, 5′-CCACATCGCTCAGACACCAT; GAPDH-RP, 5′-GGCAACAATATCCACTTTACCAGAGT; and GAPDH-probe, 5′-[6FAM]ACCAAATCCGTTGACTCCGACCTTCA[TAMRA] (all synthesized by Sigma-Aldrich, St Louis, MO, USA).

### Endocytosis assay

For analysis of the endocytic capacity, 5 × 10^4^ cells in RP10 medium were incubated in a 96-well plate with or without 0.25 mg/ml fluorescent dextran conjugate (FITC–dextran, 40,000 MW; Molecular Probes, Invitrogen, Paisley, UK) at 37°C. As a negative control, precooled cells were incubated with FITC–dextran at 4°C. After 1 hour of incubation, cells were washed for four times with phosphate-buffered saline + 0.5% bovine serum albumin and were analyzed immediately by flow cytometry.

### Quantification of nuclear factor-κB family members

Nuclear factor (NF)-κB family members (p50, p65, c-Rel, p52 and RelB) were quantified using the ELISA-based TransAM NF-κB family kit (Active Motif, Rixensart, Belgium) in whole protein lysates containing 10 μg protein per sample according to the manufacturer’s manual. Microtiter plates were analyzed with an EMax microplate reader (Molecular Devices).

### SDS-PAGE and western blotting

Whole-cell lysates were prepared using RIPA buffer containing 50 mM Tris, pH 7.4, 1% NP-40, 0.25% sodium deoxycholate, 150 mM NaCl, 1 mM ethylenediamine tetraacetic acid, 1× proteinase inhibitor (Roche, Mannheim, Germany), 1 mM phenylmethanesulfonyl fluoride, 1 mM sodium-orthovanadate and 1 mM sodium fluoride. Then 10 μg protein per lane were loaded on SDS-polyacrylamide gels and transferred onto nitrocellulose membranes. Chemicals were purchased from Sigma Aldrich (Oslo, Norway) if not otherwise stated. Membranes were probed with antibodies against Ro52/SSA (sc-25351), IRF-8 (sc-6058), Bim (sc-11425), Stat1 (sc-464), p-Stat1 Tyr701 (sc-7988), p-Stat1 Ser727 (sc-16570), Stat3 (sc-482), pStat3 (sc-8059; all antibodies purchased from Santa Cruz Biotechnology, Santa Cruz, CA, USA), and GAPDH (clone 6C5; HyTest, Turku, Finland). Horseradish peroxidase-coupled secondary antibodies were all from Bio-Rad Laboratories (Hercules, CA, USA). Proteins were visualized and quantified with a ChemiDOC XRS system and Quantity One software (Bio-Rad Laboratories) using Super Signal West Femto substrate (Pierce, Thermo Fisher Scientific, Rockford, CA, USA). Expression levels were calculated by measuring the density of the bands as a percentage in relation to a standard sample used on all blots, which was set to 100%, relative to GAPDH as loading control for each sample. The results are shown as an arbitrary unit.

### Statistical analyses

All statistical analyses were performed using Prism (GraphPad Software Inc., La Jolla, CA, USA). When necessary, datasets were analyzed with the D’Aostimo and Pearson omnibus normality test to evaluate distribution of data. Since most of the data were not normally distributed, a two-tailed nonparametric Mann–Whitney test was applied.

## Results

### Phenotypical characterization of moDC from patients with pSS compared with healthy controls

**Figure 1:**
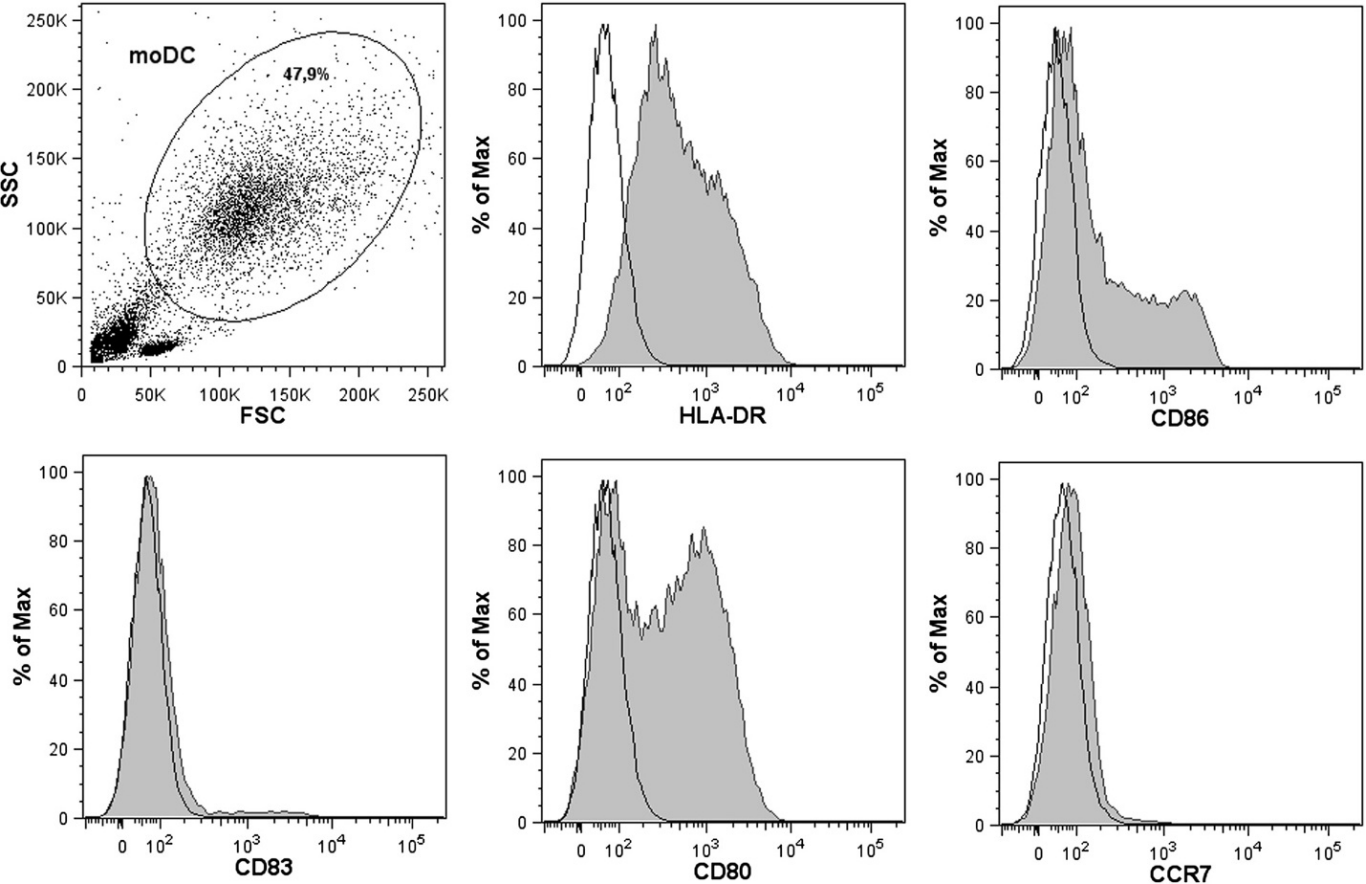
**Representative flow cytometry plots showing the monocyte-derived dendritic cell gate and histograms with isotype controls for the presented markers (HLA-DR, CD86, CD83, CD80 and CCR7).** The monocyte-derived dendritic cell (moDC) gate was set according to scatter properties. Plots show isotype control IgG (open histogram) versus specific antibody staining (grey histogram). FSC, forward scatter; SSC, side scatter.

**Figure 2:**
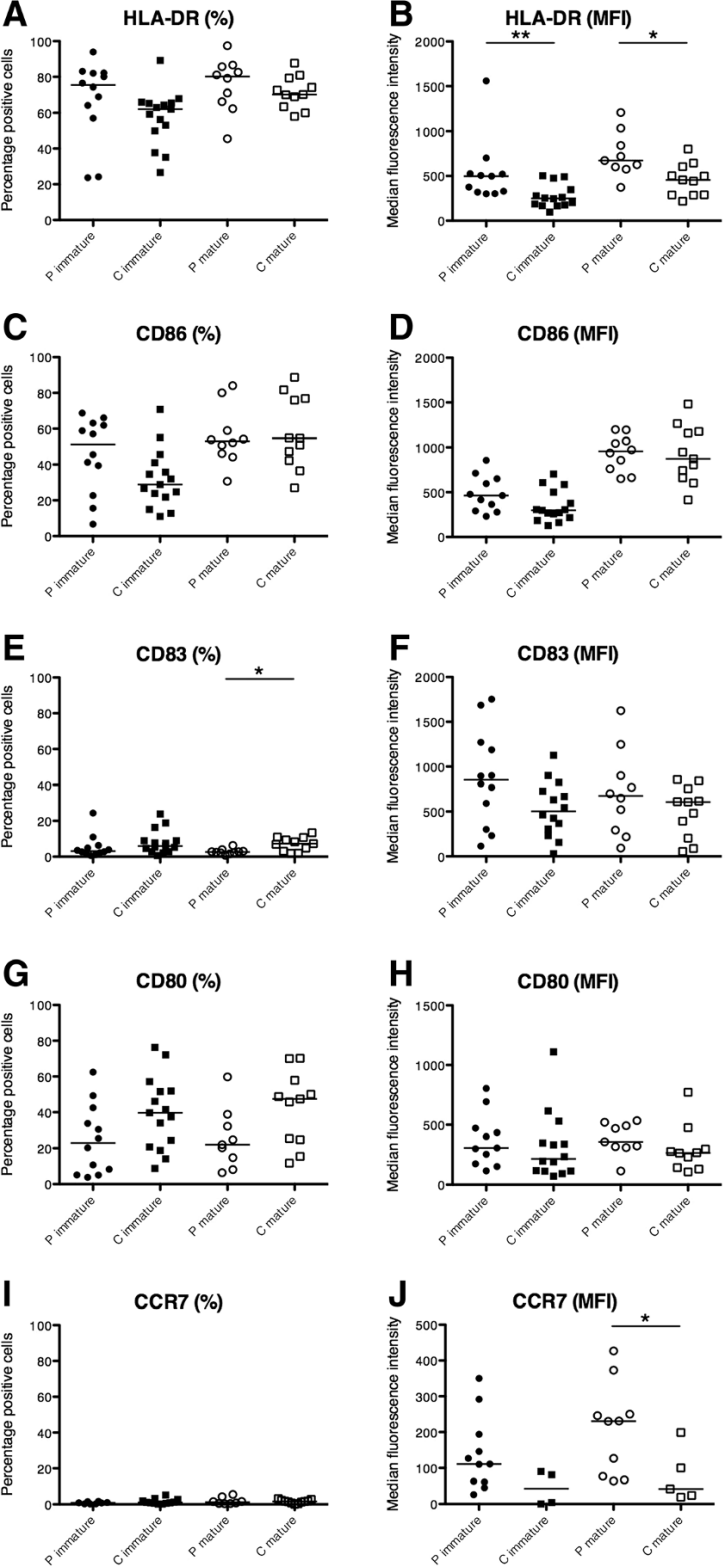
**Expression of activation markers and co-stimulatory molecules in immature and mature monocyte-derived dendritic cells from primary Sjögren’s syndrome patients and controls.** For all samples, the percentage of positive cells or the median fluorescence intensity (MFI) of the negative control was subtracted. Percentages of monocyte-derived dendritic cells (moDC) being positive for **(A)** HLA-DR, **(C)** CD86, **(E)** CD83, **(G)** CD80 and **(I)** CCR7. MFI of the corresponding moDC for **(B)** HLA-DR, **(D)** CD86, **(F)** CD83, **(H)** CD80 and **(J)** CCR7. Primary Sjögren’s syndrome patients, P immature *n* = 12 and P mature *n* = 10; controls, C immature *n* = 15 and C mature *n* = 11.

Overexpression of co-stimulatory molecules such as CD86 has been shown to affect moDC from patients with SLE, an autoimmune disorder closely related to SS [21, 22]. We therefore decided to investigate the phenotype of moDC from patients with pSS (Figures 1 and 2). Immature and mature cells from patients and healthy controls were analyzed for expression of several surface markers by flow cytometry (Figure 2). Neither the percentage of moDC positive for CD86 and CD80 (Figure 2C,G) nor expression levels represented by the MFI revealed significant variations between patients and controls (Figure 2D,H). The percentage of mature moDC being CD83-positive was higher in controls compared with patients (Figure 2E), but expression levels of CD83 were unaffected (Figure 2F). However, immature moDC and moDC matured for 48 hours from pSS patients expressed significantly higher levels of HLA-DR (Figure 2B) and CCR7 (Figure 2J).

### Apoptosis susceptibility and endocytic capacity of moDC from patients with pSS are not altered compared with healthy controls

**Figure 3:**
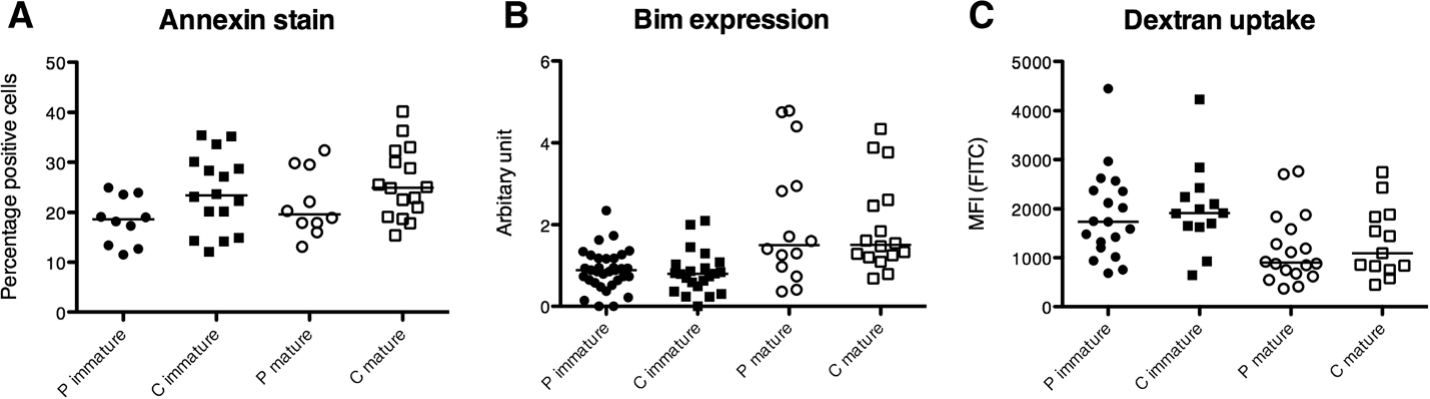
**Apoptosis susceptibility and endocytic capacity of monocyte-derived dendritic cells from patients with primary Sjögren’s syndrome and healthy controls. (A)** Percentage of monocyte-derived dendritic cells (moDC) positive for Annexin V analyzed by flow cytometry. Primary Sjögren’s syndrome patients, P immature/mature *n* = 10; controls, C immature/mature *n* = 16. **(B)** Expression of Bim in moDC samples measured by western blot. **(C)** Uptake of dextran–fluorescein isothiocyanate (FITC) by moDC. Patients, P immature *n* = 19 and P mature *n* = 18; controls, C immature *n* = 13 and C mature *n* = 13. MFI, median fluorescence intensity.

Decreased apoptosis susceptibility in moDC might lead to autoimmunity by enhancing the interaction between DC and autoreactive T cells. To examine the apoptosis susceptibility of immature and mature moDC, we stained the cells for the apoptosis marker Annexin V–FITC and the necrosis marker 7-AAD, and analyzed them by flow cytometry. We could not find differences in the amount of apoptotic cells either among immature moDC or mature moDC from pSS patients compared with controls (Figure 3A). We further analyzed the expression of the proapoptotic protein Bim in moDC by western blot. As expected, Bim expression was increased in mature moDC, but the expression did not vary between moDC from patients and controls (Figure 3B). We also compared the endocytic capacity of immature and TLR7/8-matured moDC from pSS patients and controls measuring the uptake of FITC-labeled dextran by flow cytometry without finding significant differences (Figure 3C).

### Levels of TLR7 do not vary between moDC from pSS patients and healthy controls

**Figure 4:**
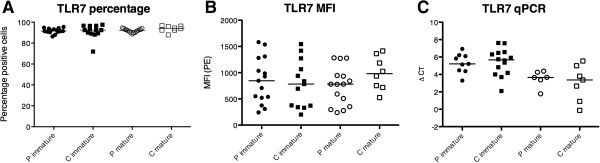
**TLR7 expression in immature and mature monocyte-derived dendritic cells from primary Sjögren’s syndrome patients and controls. (A)** Percentage of monocyte-derived dendritic cells (moDC) positive for toll-like receptor 7 (TLR7) analyzed by flow cytometry. Primary Sjögren’s syndrome (pSS) patients, P immature *n* = 18 and P mature *n* = 16; controls, C immature *n* = 16 and C mature *n* = 11. **(B)** Median fluorescence intensity (MFI) of TLR7 in the corresponding moDC samples. **(C)** TLR7 on transcriptional level analyzed by real-time quantitative polymerase chain reaction (qPCR) in moDC from pSS patients and controls. pSS, P immature *n* = 9 and P mature *n* = 6; controls, C immature *n* = 14 and C mature *n* = 7. PE, phycoerythrin.

Since we used a TLR7/8 ligand to generate mature moDC from pSS patients and controls, we analyzed TLR7 expression to exclude that possible variations in TLR7 expression levels would influence our results. Using flow cytometry, we did not detect significant differences in percentage of positive cells or MFI of TLR7 in immature moDC and moDC matured for 48 hours between patients and controls (Figure 4A,B). To further validate TLR7 levels on a transcriptional level, we extracted RNA from moDC and performed real-time quantitative PCR using TLR7-specific primers and probe. Even though mature cells had reduced amounts of TLR7 mRNA, levels of TLR7 transcripts did not vary between immature and mature moDC from pSS patients compared with controls (Figure 4C).

### Levels of secreted cytokines by moDC from patients with pSS differ significantly from healthy controls

Cytokine imbalances are often discussed in the context of autoimmunity. To study whether these cytokines might derive from moDC, we analyzed undiluted cell culture supernatants from immature and matured moDC by ELISA and 25-plex Luminex analyses. The concentration of most cytokines was generally low, apart from IL-4 and GM-CSF that were added to the cell culture to generate moDC. We therefore reanalyzed IL-4 and GM-CSF levels in diluted samples by ELISA, finding significantly lower levels of IL-4 in immature pSS samples compared with immature control samples (mean ± standard deviation (pg/ml): P immature, 11,595.9 ± 3,127.1; C immature, 9,590.8 ± 1,512.6). Using 25-plex Luminex analyses, we detected significant different levels of IL-7, IL-12p40, TNFα, IFN gamma and MIP-1α in mature moDC from pSS patients compared with controls (Table 2). We found significantly decreased levels of the chemokine of IL-7 and IFN gamma in mature moDC from pSS patients compared with controls. Moreover, we found that mature moDC from patients produced significant higher levels of IL-12p40, TNFα and MIP-1α compared with mature moDC from healthy controls. We further tested the biologically active forms of IL-12p40, IL-12p70 and IL-23 by ELISA, but IL-23 was not secreted in detectable amounts while IL-12p70 was secreted only by mature moDC without significantly altered levels (mean ± standard deviation (pg/ml): P mature, 48.8 ± 68.7; C mature, 44.9 ± 82.0). BAFF, a cytokine that has been found to be upregulated in plasma from patients with pSS [28], was measured in moDC supernatants, showing that immature and mature moDC from pSS patients secreted higher amounts of BAFF compared with moDC from controls, but without reaching statistical significance (mean ± standard deviation (pg/ml): P immature, 41.8 ± 25.0; C immature, 31.3 ± 18.1; P mature, 38.5 ± 28.5; C mature, 28.6 ± 26.2). We also measured levels of multiple subtypes of IFN alpha in cell culture supernatants by ELISA as IFNs play a crucial role in the development of autoimmune diseases, without finding significant amounts being secreted by moDC (data not shown).

**Table 2:** **Comparison of cytokines secreted by immature or TLR7/8 stimulated mature moDC from pSS patients and controls analyzed by multiplex Luminex analyses**

Cytokine (pg/ml)	Patients immature (*n* = 21)	Controls immature (*n* = 17)	Patients mature (*n* = 20)	Controls mature (*n* = 16)
IL-7	30.9 ± 11.0	35.3 ± 29.8	31.4 ± 11.0	40.3 ± 16.2*
IL-12p40	703.9 ± 878.8	498.9 ± 362.8	3847.9 ± 5867.8	796.3 ± 326.8**
IL-17	86.0 ± 22.1	83.2 ± 31.9	76.6 ± 15.1	101.5 ± 39.4*
TNFα	28.9 ± 22.1	24.3 ± 8.8	53.9 ± 20.5	39.8 ± 17.9*
Interferon gamma	18.3 ± 7.9	24.1 ± 12.5	20.5 ± 8.9	29.1 ± 9.3**
MIP-1α	298.5 ± 138.8	304.3 ± 146.5	398.7 ± 201.3	304.6 ± 228.5*

Only cytokines with a significant different value are shown. Data presented as mean ± standard deviation. IL, interleukin; MIP, macrophage inflammatory protein; moDC, monocyte-derived dendritic cells; pSS, primary Sjögren’s syndrome; TLR, toll-like receptor; TNFα, tumor necrosis factor alpha. A significant difference (*P < 0.05 and **P < 0.01) for the levels of six different cytokines was found when comparing mature moDC from pSS patients with mature moDC from healthy controls.

### Elevated levels of activated RelB correlate with increased IL-12p40 secretion

**Figure 5:**
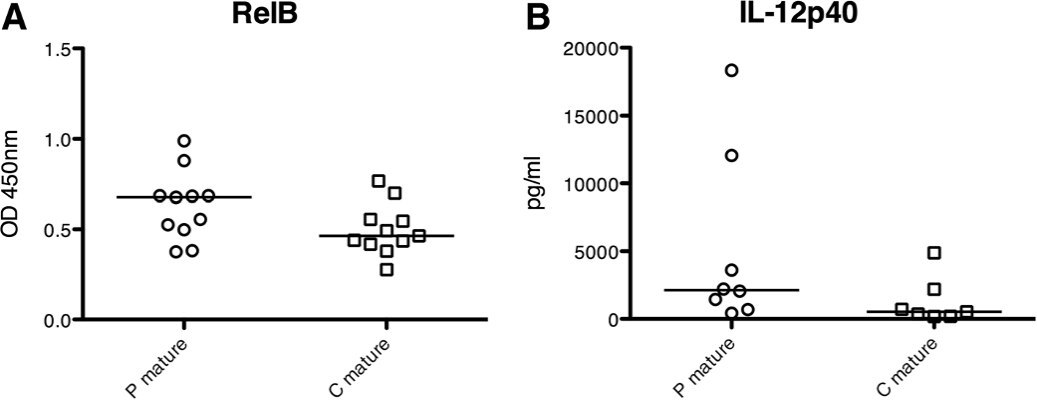
**Expression of activated RelB correlates to elevated secretion of IL-12p40. (A)** Levels of RelB measured by the enzyme-linked immunosorbent assay (ELISA)-based TransAM nuclear factor-κB family kit are slightly increased in mature monocyte-derived dendritic cells from primary Sjögren’s syndrome patients (P mature, *n* = 11) compared with healthy controls (C mature, *n* = 11). **(B)** IL-12p40 secretion quantified by ELISA in the same, corresponding samples is increased. OD, optical density.

To investigate possible factors involved in increased cytokine secretion of moDC from pSS patients stimulated for 48 hours with a TLR7/8 ligand, we quantified activated NF-κB family members (p50, p65, c-Rel, p52 and RelB) using the ELISA-based TransAM NF-κB family kit. We found that activated RelB is increased in mature moDC from patients with pSS (Figure 5A), while none of the other NF-κB family members being upregulated or downregulated (data not shown). IL-12p40 secretion was reanalyzed for the corresponding samples and quantified by ELISA. The result confirmed previous multiplex data and revealed increased levels in mature moDC from pSS patients (Figure 5B), without reaching the statistical significance seen in multiplex analyses due to smaller sample size. Nevertheless, samples with highest RelB expression correlate to samples with highest IL-12p40 secretion.

### Stat1 expression is significantly reduced in mature moDC from patients with pSS compared with healthy controls

We next aimed to investigate protein expression levels of the SS-related autoantigen Ro52/SSA in moDC, because increased mRNA levels of Ro52/SSA were found in peripheral blood mononuclear cells from patients with pSS. We also analyzed the expression levels of IRF-8, a potential downstream target of Ro52/SSA, in moDC from patients with pSS compared with healthy controls by western blot. No differences in Ro52/SSA and IRF-8 protein expression in whole cell lysates from moDC between pSS patients and healthy controls could be observed (data not shown).

**Figure 6:**
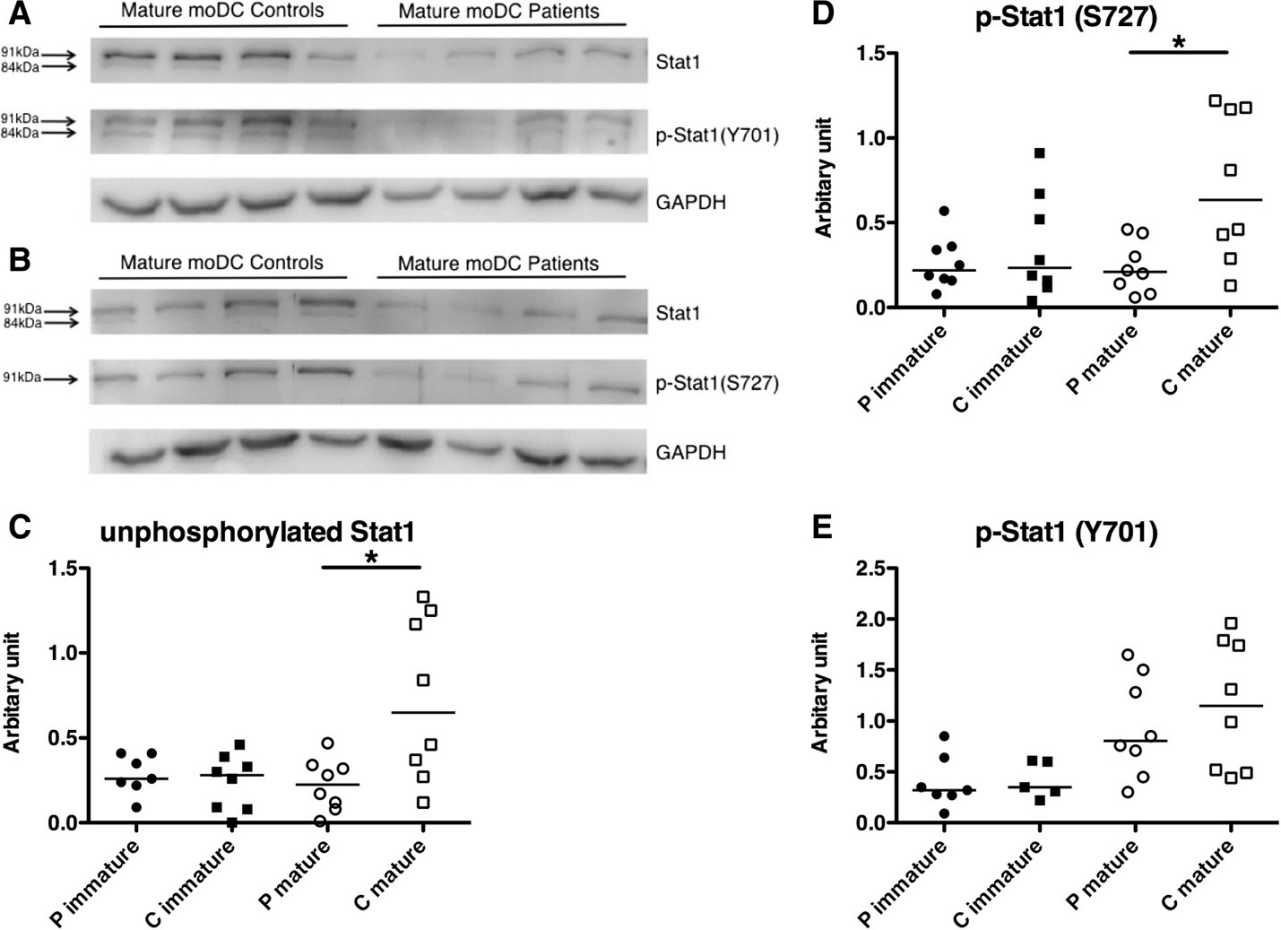
**Stat1 expression is significantly reduced in TLR7/8 stimulated monocyte-derived dendritic cells from primary Sjögren’s syndrome patients. (A)**, **(B)** Representative western blots for Stat1α, p-Stat1α (Y701), p-Stat1α (S727) and glyceraldehyde 3-phosphatase dehydrogenase (GAPDH) expression in mature monocyte-derived dendritic cells (moDC) from primary Sjögren’s syndrome (pSS) patients (*n* = 4) and controls (*n* = 4). **(C)** Stat1 expression in immature and mature moDC from patients (*n* = 8) and controls (*n* = 8). **(D)** p-Stat1α (S727) expression in immature and mature moDC from patients (*n* = 8) and controls (*n* = 8). **(E)** p-Stat1α (Y701) expression in immature and mature moDC from patients (P immature *n* = 7 and P mature *n* = 8) and controls (C immature *n* = 5 and C mature *n* = 8). Relative expression levels are presented as arbitrary units and were calculated as the percentage of band density normalized to GAPDH expression in relation to a standard sample used on all blots (set to 100%).

To investigate possible altered signaling molecules on protein level in moDC from pSS patients, we decided to focus on members of the Stat signal transducer and transcription factor family, as they are crucial for IFN signaling. We compared unphosphorylated Stat1 and Stat3 as well as phosphorylated p-Stat1 (Y701), p-Stat1 (S727) and p-Stat3 (Y705) in protein lysates from immature and mature moDC. No differences in Stat3 and p-Stat3 levels were detected in protein lysates of mature moDC from patients and controls (data not shown). However, we found significantly reduced expression of Stat1α (91 kDa) in mature moDC from patients with pSS (Figure 6C), representative blots shown in Figure 6A,B. Stat1β (84 kDa) was generally expressed at low levels, making a proper quantification difficult. We therefore restricted further analyses to the full-length 91 kDa Stat1α isoform. We also analyzed the amounts of phosphorylated Stat1 levels, and detected significantly less p-Stat1 (S727) (Figure 6D) and reduced p-Stat1 (Y701) (Figure 6E) in mature moDC from pSS patients. Furthermore, we analyzed both IRF-1 and IRF-9 on a transcriptional level by real-time quantitative PCR, because they are downstream targets of the Stat1 signaling cascade, without finding significant differences (data not shown).

## Discussion

In this study, we analyzed phenotypical and functional properties of moDC from patients with pSS and compared them with gender-matched and age-matched healthy controls to elucidate whether DC might be involved in the pathogenesis of SS. We discovered that moDC from pSS patients express significantly increased levels of HLA-DR and CCR7 and that mature moDC from pSS patients secrete altered amounts of several cytokines. Most of the analyzed cytokines were detected at low levels, but two of them (IL-12p40 and MIP-1α) were found in comparable amounts also in serum samples from pSS patients [29]. Moreover, we found that increased IL-12p40 correlated with increased amounts of nuclear RelB, indicating an overactivated NF-κB pathway in mature moDC from pSS patients. Furthermore, we found that moDC from pSS patients matured for 48 hours with a TLR7/8 ligand express significantly less Stat1 compared with cells from healthy controls. Stat1 is a signal transducer and transcription factor, which is especially important for the responsiveness to IFN, but was also found to act as a suppressor of NF-κB activation [30]. Decreased levels of Stat1 in mature moDC from pSS patients might thus cause higher RelB activation and eventually lead to elevated cytokine production.

Under inflammatory conditions, monocytes can turn into inflammatory moDC, which can be generated *in vitro* serving as an appropriate model for studying DC [31]. Migration of monocytes to the salivary glands, where they turn into inflammatory moDC, has been proposed as a possible source for DC found in the glands from SS patients [32]. The increase in CCR7 on mature moDC from pSS patients might indicate that the homing to the lymph node of these cells in response to the CCR7 ligands CCL19 or CCL21 can be enhanced, but the general expression of CCR7 is relatively low, thus making a valid interpretation rather difficult.

Immature DC are reckoned to mediate tolerance [33] and overactive or preactivated DC have been suggested to be involved in the development of autoimmune diseases [34] via augmented activation of autoreactive T cells instead of anergy induction. Overexpression of co-stimulatory molecules such as CD86 correlating with increased disease activity has been described previously in moDC of patients with SLE (reviewed in [35]). We found increased levels of HLA-DR but not CD86 (Figure 2B,D), clearly distinguishing moDC of SS patients from those of SLE patients. An increase in HLA-DR expression indicates that moDC from pSS patients are more active in antigen presentation than moDC from controls, which might be due to the fact that they derive from an inflammatory environment. Interestingly, a strong association of SS with genes encoding for HLA molecules has been reported, implicating a genetic predisposition being associated with the disease SS [36], and thus stressing the importance of performing further studies on antigen-presenting functions in these patients.

We also focused on cytokine profiles of immature and mature moDC from pSS patients and controls and analyzed the cell culture supernatants by multiplex Luminex analyses (Table 2). DC might be a possible source for cytokines identified in serum from patients with pSS. We detected significantly higher amounts of the proinflammatory cytokines IL-12p40, TNFα, and MIP-1α in supernatants of mature moDC from pSS patients. Both IL-12p40 and MIP-1α levels correspond to amounts that have been found in sera of pSS patients [29]. IL-12 is produced by DC upon activation [37]. The biological active form of IL-12 is a heterodimeric protein (IL-12p70) consisting of two subunits, IL-12p40 and IL-12p35, directing immune responses towards Th1 cells. IL-12p40 can also be part of IL-23 as a heterodimer with the IL-23p19 subunit. We found significantly more IL-12p40 in supernatants from TLR7/8-stimulated moDC from pSS patients compared with controls, but we could not detect IL-23 in notable amounts. IL-12p70 was secreted in small amounts by mature moDC, but without significant differences between moDC from pSS patients compared with controls (data not shown). Interestingly, a previous study reported elevated serum titers of IL-12p40 in SS patients compared with controls [38] and in comparable amounts with those measured in this study. DC might therefore be the IL-12p40-producing cells in patients with pSS. Although the roles of the biological active forms of IL-12p40 are better known, it was shown recently that IL-12p40 homodimers attenuate regulatory T cells in mice [39]. Increased IL-12p40 levels in pSS patients might therefore influence the function of regulatory T cells and consequently prevent peripheral tolerance due to suppression of regulatory T cells. Apart from elevated IL-12p40 and MIP-1α levels, we detected significantly increased levels of TNFα and decreased levels of IL-7 and IFN gamma in mature moDC of pSS patients. Nevertheless, the amounts of those cytokines were quite low in general, making the significance of the results difficult to discuss. We also detected significantly reduced IL-4 levels in immature pSS samples, but we cannot conclude whether this difference occurred due to different secretion or consumption of moDC from pSS patients because IL-4 was added to the cell culture.

To investigate signaling pathways being involved in altered cytokine secretion of moDC from pSS patients stimulated for 48 hours with a TLR7/8 ligand, we quantified the activated NF-κB family members p50, p65, c-Rel, p52 and RelB. Association of NF-κB genes with a positive autoantibody titer was found in patients with pSS previously [40]. Here we detected that expression of activated RelB was increased in moDC from pSS patients. Samples with the highest RelB expression correlated to samples with the highest IL-12p40 secretion, pointing in the direction of enhanced signaling via an activated NF-κB pathway.

To further investigate possible altered signaling molecules in moDC from pSS patients, we investigated members of the Stat signal transducer and transcription factor family. The functions of activated Stat1 proteins are complex since Stat1 drives expression of multiple genes, with a key role in IFN signaling (reviewed in [41]). Although the IFN signature seen in patients with pSS is rather associated with plasmacytoid DC, systemic increase in type I IFN might affect Stat1 pathways in other immune cells such as moDC. Stat1 proteins become activated via phosphorylation on tyrosine (Y701) to initiate dimerization and serine (S727) to fully develop transcriptional activity in the nucleus. We found significantly lower protein levels of Stat1α and p-Stat1 (S727) and lowered amounts of p-Stat1 (Y701) and a visible reduction of Stat1β in mature moDC from patients with pSS. Stat1 can act as a suppressor of NF-κB activation [30] and therefore an increase in activated RelB and IL-12p40 secretion can be a consequence of altered Stat1 signaling, which might develop under the influence of IFN. Because we used a TLR7/8 ligand to stimulate the moDC, we analyzed levels of TLR7 by flow cytometry and real-time quantitative PCR. No differences were observed and thus our findings cannot be explained by alterations in levels of TLR7.

This study emphasizes the importance of focusing on studies of molecular mechanisms at the cellular level, which indicate a possible dysfunction of DC contributing to the inflammatory environment seen in autoimmunity. Because Stat1 mRNA levels were not altered when tested using real-time quantitative PCR, the mechanisms behind the regulation of Stat1 proteins in mature moDC need to be further investigated. It is of particular interest to further elucidate the relation between Stat1 and the IFN signature seen in patients with pSS by including the analyses of plasmacytoid DC in these studies. Besides analyzing different time points after DC stimulation, further studies should include different stimuli, as our study is limited to the TLR7/8 ligand CL097.

## Conclusions

Our data reveal a distinct decrease in Stat1 protein levels in mature moDC from patients with pSS, possibly leading to increased activated nuclear RelB and consequently elevated IL-12p40 secretion. These results indicate a defect of mature moDC after activation by TLR7/8 ligand. Furthermore, we found that immature and mature moDC from pSS patients express more HLA-DR molecules compared with healthy controls, a result that might build a functional link to the genetic association with HLA-DR genes seen in pSS patients. Expression of CCR7 is increased in mature moDC from pSS patients, which could lead to an increased homing ability of these cells. Since this study is covering only a limited number of functional analyses, we cannot totally rule out deficiencies in other DC functions. In conclusion, we suggest that activated moDC influence the pathogenesis of pSS via cytokine production and propose further studies on Stat1 signaling, Stat1 regulating mechanisms and antigen-presenting functions to further elucidate the cause and function of decreased Stat1 and increased HLA-DR molecules.

## Abbreviations

APC: antigen-presenting cell
BAFF: B-cell activating factor
DC: dendritic cells
ELISA: enzyme-linked immunosorbent assay
FITC: fluorescein isothiocyanate
GAPDH: glyceraldehyde 3-phosphatase dehydrogenase
GM-CSF: granulocyte–macrophage colony-stimulating factor
IFN: interferon
IL: interleukin
IRF: interferon-regulated factor
MFI: median fluorescence intensity
MIP: macrophage inflammatory protein
moDC: monocyte-derived dendritic cells
NF: nuclear factor
PE: phycoerythrin
pSS: primary Sjögren’s syndrome
PCR: polymerase chain reaction
SLE: systemic lupus erythematosus
Stat: signal transducer and activator of transcription
TLR: toll-like receptor
TNFα: tumor necrosis factor alpha.
